# Prenatal Opioid Exposure and Immune-Related Conditions in Children

**DOI:** 10.1001/jamanetworkopen.2023.51933

**Published:** 2024-01-17

**Authors:** Erin Kelty, Kaitlyn Rae, Lauren L. Jantzie, Caitlin S. Wyrwoll, David B. Preen

**Affiliations:** 1 School of Population and Global Health, The University of Western Australia, Crawley, Western Australia, Australia; 2 School of Human Sciences, The University of Western Australia, Crawley, Western Australia, Australia; 3 Division of Neonatal-Perinatal Medicine, Department of Pediatrics, Johns Hopkins University School of Medicine, Baltimore, Maryland

## Abstract

**Importance:**

Prenatal opioid exposure (POE) may alter with fetal development of the immune system, which may influence long-term health and susceptibility to immune-related conditions.

**Objective:**

To compare the risk of hospitalization and emergency department presentation for immune-related conditions in children with and without POE.

**Design, Setting, and Participants:**

This retrospective, population-based cohort study used linked administrative health records of all children born in Western Australia between January 1, 2003, and December 31, 2018 (N = 401 462).

**Exposure:**

Prenatal exposure to prescription opioids (overall and by trimester), neonatal abstinence syndrome diagnosis, and opioid indication (pain or opioid use disorder [OUD]).

**Main Outcomes and Measures:**

The main outcome was hospital admissions and emergency department presentations during which a child was diagnosed with an immune-related condition, including infections, conditions associated with an overactive immune system (eg, asthma, eczema, and allergy and anaphylaxis), and autoimmune diseases diagnosed before age 5 years or June 30, 2020. Data were analyzed between August 30, 2022, and February 27, 2023.

**Results:**

Neonates with POE (1656 [0.4%]; mean [SD] gestational age, 37.7 [2.1] weeks; 836 females [50.5%]; 820 males [49.5%]) were more likely to be born preterm, have low birth weight for gestational age, and be coexposed to cigarette smoke compared with nonexposed neonates. Perinatal opioid exposure was associated with an increased risk of perinatal infection (adjusted odds ratio [AOR], 1.62; 95% CI, 1.38-1.90) and eczema and dermatitis (AOR, 11.91; 95% CI, 9.84-14.41) compared with nonexposure. Neonatal abstinence syndrome was also associated with both conditions (AOR, 2.91 [95% CI, 2.36-3.57] and 31.11 [95% CI, 24.64-39.28], respectively). Prenatal opioid exposure was also associated with an increased risk of childhood asthma (adjusted hazard ratio [AHR], 1.44; 95% CI, 1.16-1.79), but not allergies and anaphylaxis. It was also associated with an increased risk of childhood eczema and dermatitis, but only in children with POE from opioids used to treat OUD (AHR, 1.47; 95% CI, 1.08-1.99) rather than pain. In contrast, POE from opioids used for pain was associated with an increased risk of infection (AHR, 1.44; 95% CI, 1.32-1.58), but POE to opioids used to treat OUD was not. Autoimmune conditions were rare and were not observed to be associated with POE.

**Conclusions and Relevance:**

In this cohort study, POE was associated with an increased risk of infection, eczema and dermatitis, and asthma, but not allergies and anaphylaxis or autoimmune conditions. These findings highlight the importance of further study of opioid-induced immune changes during pregnancy, the potential impact on long-term health in exposed children, and the mechanisms of opioid-induced immune dysregulation.

## Key Points

**Question:**

What is the association between prenatal opioid exposure (POE) and risk of immune-related conditions?

**Findings:**

In this cohort study of 401 462 neonates, POE was associated with an increased odds of infections and of eczema and dermatitis during the perinatal period. Beyond the perinatal period, POE was associated with an increased risk of infections, asthma, and eczema and dermatitis but not with allergies and anaphylaxis or autoimmune conditions.

**Meaning:**

These findings suggest that, consistent with animal studies, POE may have a long-term impact on the immune system and child health.

## Teaser

This cohort study compares the risk of hospitalization and emergency department visits for immune-related conditions among children with prenatal opioid exposure.

## Introduction

Opioids play a critical role in fetal development.^1,2,3^ The use of exogenous opioids during pregnancy has the potential to interfere with these finely regulated physiologic processes, affecting a wide range of body systems,^4,5^ including the immune system.^6,7,8,9,10^ Emerging evidence in rodent models has suggested that opioid exposure during critical periods of fetal immune development may result in immune priming, such that the immune system overreacts to subsequent and later immune activation.^6,11^ Clinically, evidence of the influence of prenatal opioid exposure (POE) on the immune system has been observed in umbilical cord samples, with exposed neonates having lower levels of neutrophils, inflammatory cytokines, and chemokines compared with nonexposed neonates, indicative of dysregulated inflammatory processes.^12^

Opioids have been observed to be immunosuppressive in adults,^13^ with increased infection rates, particularly pneumonia, observed in patients treated with opioids for pain.^14^ However, clinical data suggest that not all opioids have an effect on the immune system, with opioids broadly classified as immunosuppressive (eg, codeine, morphine, fentanyl, and methadone) or nonimmunosuppressive (eg, oxycodone, buprenorphine, and tramadol).^14,15^ At present, the mechanism behind why some opioids are more likely to be associated with immune-related conditions is poorly understood. Given the differences in immunosuppressive qualities of opioids in adults, the same may also be true for POE, although this remains a substantial gap in the current literature.

In addition to opioid exposure itself, opioid withdrawal may also negatively affect the adult immune system. In nonpregnant mice, withdrawal from morphine has been shown to be immunosuppressive, with morphine withdrawal associated with reduced survival time and an increased *Salmonella* burden in mice exposed to oral infection.^16^ Similarly, mice withdrawn from morphine are more susceptible to sublethal doses of bacterial lipopolysaccharide.^17^ These findings are clinically interesting because not all neonates with POE develop a withdrawal syndrome (ie, neonatal abstinence syndrome [NAS] or neonatal opioid withdrawal syndrome) following birth^18^; thus, the association between POE and immune-related conditions may be modified by the presence of withdrawal.

Preliminary evidence has suggested that POE may contribute to a child’s susceptibility to immune-related conditions. However, the potential effects may rely on the timing of exposure, corresponding with critical windows of vulnerability in development of the immune system,^19^ the presence or absence of neonatal opioid withdrawal syndrome, and the type of opioid exposure, but limited data exist. Additionally, research thus far has focused predominantly on infections, with an absence of research on other immune-related conditions.

This study examined the association between POE and immune-related conditions in childhood. Furthermore, the study aimed to examine whether the association between POE and immune-related conditions differs based on the timing of exposure (trimester), the indication for opioid dispensing, or the presence of NAS.

## Methods

This retrospective cohort study was approved by the human research ethics committees of the Western Australia Department of Health (RGS0000003029) and The University of Western Australia (RA/4/20/5530). A waiver of informed consent was approved for the project based on the criteria outlined in the *National Statement on Ethical Conduct in Human Research*.^20^ The study followed the Strengthening the Reporting of Observational Studies in Epidemiology (STROBE) reporting guideline.

The study included all live-born children born in Western Australia between January 1, 2003, and December 31, 2018, with data linked from state hospital, emergency department (ED), and mortality records. Children were identified from the Midwives Notification System (MNS), which includes all neonates born in Western Australia.

### Outcomes

Outcomes were largely grouped into 3 types of immune system–related conditions. The first included infective diseases, overall and by type-specific conditions (as outlined by Videholm et al^21^). The second included conditions associated with an overactive immune system and inflammation, such as asthma, eczema, and allergies and anaphylaxis. The third included autoimmune diseases, such as rheumatoid conditions (juvenile idiopathic arthritis and systemic lupus erythematosus), inflammatory bowel disease, and type 1 diabetes.

### Data Linkage and Data Sources

Eligible pregnant women and their children were identified within the MNS. For the children, data were extracted from the Hospital Morbidity Data Collection, the ED Data Collection, and the Western Australia Death Register. For pregnant women, data were obtained from the Monitoring of Drugs of Dependence System, which contains information on the dispensing of all Schedule 8 medicines in Western Australia, including methadone, buprenorphine, oxycodone, fentanyl, morphine tapentadol, pethidine, hydromorphine, and high-dose codeine. This system does not capture the dispensing of lower schedule opioids, including low-dose or combination codeine preparations and tramadol. Neonates were classified as having POE if 2 or more prescriptions, totaling 40 U or more, were dispensed during pregnancy as determined from their date of birth and estimated length of gestation in the MNS.

Linked hospital and ED data were used to identify the diagnoses of conditions of interest. For each hospital separation, a primary diagnosis and up to 20 contributing codiagnoses could be recorded. For ED presentations, only a single diagnosis was assigned. Mortality data were used to assist with censoring of follow-up time. The *International Statistical Classification of Diseases and Related Health Problems, Tenth Revision, Australian Modification* was used to assign diagnoses for both the hospital and ED data. For infection, diagnoses were derived from Videholm et al,^21^ while codes for autoimmune conditions were compiled from multiple published studies.^22,23,24,25^

### Statistical Analysis

Perinatal conditions (infections and eczema and dermatitis diagnosed within 28 days of birth) were analyzed using logistic regression. Univariable and multivariable models were used, with multivariable models adjusted for preterm birth (<37 weeks’ gestation), low birth weight for gestational age (<10th percentile), low Apgar score (<7), resuscitation at birth (yes, no), prelabor rupture of membranes, and socioeconomic status. Socioeconomic status was estimated based on the Socio-Economic Indexes for Areas Index of Relative Socio-Economic Advantage and Disadvantage ranking, calculated based on statistical area, level 1 for the address given at birth. Perinatal conditions were examined separately by each trimester of exposure, opioid exposure with an NAS diagnosis, maternal exposure to opioids used to treat pain, exposure to opioids used to treat opioid use disorder (OUD), and nonexposure. Where models of POE with an NAS diagnosis were associated with odds ratios (ORs) higher than opioid exposure alone, additional analysis was conducted using both POE and NAS in the model.

Childhood conditions (infections, asthma, eczema and dermatitis, and allergies and anaphylaxis diagnosed from age 29 days to 5 years) were analyzed using univariable and multivariable Cox proportional hazards regression models. The multivariable models were adjusted for maternal age at birth, sex of the child, cesarean delivery, smoking status during pregnancy, preterm birth, and socioeconomic status. Race and ethnicity data were not provided to the research team for this study. These data are not well collected in the available data sets, and there are some barriers with the presentation and use of Aboriginality in Australian data. Survival data were censored at the first event, child’s 5th birthday, death at age younger than 5 years, or the censoring date of June 30, 2020. Per perinatal conditions, childhood conditions were also examined by trimester, NAS diagnosis, and opioid indication. Infection diagnoses were further examined by infection subtypes. The analysis was performed from August 30, 2022, to February 27, 2023, using Stata/MP, version 16 statistical software (StataCorp LLC). A 2-sided *P* < .05 was considered significant.

## Results

The study consisted of 401 462 children, including 1656 with POE (0.4%; mean [SD] gestational age, 37.7 [2.1] weeks; 836 females [50.5%]; 820 males [49.5%]) and 399 806 without POE (99.6%; mean [SD] gestational age, 38.6 [1.9] weeks; 48.7% female; 51.3% male). All children had a minimum follow-up of 1.5 years, with a mean (SD) follow-up of 4.9 (0.4) years for both groups. Children with POE were more likely to have been born preterm (18.7% vs 8.4%) and at a low birth weight for gestational age (15.1% vs 8.0%) compared with children without POE (Table 1). Additionally, pregnant women who had used prescription opioids during pregnancy were more likely to have smoked during pregnancy (57.0% vs 11.9%) and to have previously been pregnant (87.4% vs 71.7%) compared with those who did not take prescription opioids during pregnancy. Of the children with POE, 1172 (70.8%) were exposed during trimester 1, 1220 (73.7%) during trimester 2, and 1265 (76.4%) during trimester 3. Neonatal abstinence syndrome was diagnosed in 666 children (40.2%), and 679 children (41.0%) had POE associated with an opioid used for the treatment of pain, while 1007 (60.8%) were exposed to an opioid used for the treatment of OUD (Table 2).

**Table 1.** Characteristics of Neonates and Their Mothers, Stratified by POE

Characteristic	No. (%)		*P* value
	POE	No POE	
No. of neonates	1656	399 806	NA
Gestational age, mean (SD), wk	37.7 (2.1)	38.6 (1.9)	<.001
Sex			
Female	836 (50.5)	194 538 (48.7)	.14
Male	820 (49.5)	205 268 (51.3)	
Preterm birth	310 (18.7)	33 385 (8.4)	<.001
Low birth weight for gestational age	250 (15.1)	31 984 (8.0)	<.001
Low Apgar score (<7) at 5 min	42 (2.5)	5672 (1.4)	<.001
Resuscitation	670 (40.5)	106 687 (26.7)	<.001
Prelabor rupture of membranes	97 (5.9)	15 923 (4.0)	<.001
Previous pregnancy	1447 (87.4)	286 765 (71.7)	<.001
Smoking during pregnancy	944 (57.0)	47 675 (11.9)	<.001
Maternal age, mean (SD), y	30.8 (5.4)	30.4 (5.6)	.006
Cesarean delivery	642 (38.8)	144 806 (36.2)	.03
Rurality			
Major city	1118 (67.6)	263 729 (66.0)	.02
Inner regional	332 (20.1)	72 033 (18.0)	
Outer regional	120 (7.3)	30 136 (7.5)	
Remote	43 (2.6)	17 454 (4.4)	
Very remote	41 (2.5)	16 180 (4.1)	
Socioeconomic status			
1 (Low)	226 (13.7)	31 995 (8.0)	<.001
2	258 (15.6)	55 122 (13.8)	
3	563 (24.0)	124 996 (31.3)	
4	386 (23.3)	103 (26.0)	
5 (High)	221 (13.4)	83 495 (20.9)	

Abbreviations: NA, not applicable; POE, prenatal opioid exposure.

**Table 2.** Perinatal Infection and Eczema and Dermatitis Outcomes in Neonates With POE in Western Australia, 2003-2018

	No. (%)	Infection, No. (%)	OR (95% CI)^a^	*P* value	AOR (95% CI)^b^	*P* value
**Perinatal infections**						
No POE	399 806	22 102 (5.5)	NA	NA	NA	NA
Any POE	1656	182 (11.0)	2.11 (1.81-2.46)	<.001	1.62 (1.38-1.90)	<.001
Trimester 1 POE	1172 (70.8)	133 (11.4)	2.19 (1.83-2.62)	<.001	1.68 (1.39-2.03)	<.001
Trimester 2 POE	1220 (73.7)	141 (11.6)	2.23 (1.87-2.66)	<.001	1.66 (1.38-2.00)	<.001
Trimester 3 POE	1265 (76.4)	142 (11.2)	2.27 (1.91-2.71)	<.001	1.81 (1.51-2.17)	<.001
POE + NAS	666 (40.2)	122 (18.3)	3.83 (3.15-4.67)	<.001	2.91 (2.36-3.57)	<.001
POE pain	679 (41.0)	55 (8.1)	1.51 (1.14-1.99)	.004	1.10 (0.82-1.46)	.53
POE OUD	1007 (60.8)	130 (12.9)	2.53 (2.11-3.05)	<.001	1.99 (1.64-2.41)	<.001
**Perinatal eczema and dermatitis**						
No POE	399 806	2478 (0.6)	NA	NA	NA	NA
Any POE	1656	154 (9.3)	16.44 (13.86-19.50)	<.001	11.91 (9.84-14.41)	<.001
Trimester 1 POE	1172 (70.8)	125 (10.7)	19.14 (15.84-23.14)	<.001	14.63 (11.40-18.13)	<.001
Trimester 2 POE	1220 (73.7)	132 (10.8)	19.45 (16.17-23.40)	<.001	13.61 (11.04-16.78)	<.001
Trimester 3 POE	1265 (76.4)	137 (10.8)	20.73 (17.28-24.87)	<.001	15.35 (12.50-18.84)	<.001
POE + NAS	666 (40.2)	123 (18.5)	36.32 (29.75-44.35)	<.001	31.11 (24.64-39.28)	<.001
POE pain	679 (41.0)	36 (5.3)	8.98 (6.40-12.59)	<.001	5.11 (3.54-7.38)	<.001
POE OUD	1007 (60.8)	126 (12.5)	22.93 (18.95-27.75)	<.001	19.08 (15.34-23.72)	<.001

Abbreviations: AOR, adjusted odds ratio; NA, not applicable; NAS, neonatal abstinence syndrome; OR, odds ratio; OUD, opioid use disorder; POE, prenatal opioid exposure.

^a^ Compared with no exposure.

^b^ Adjusted for preterm birth, low birth weight for gestational age, Apgar score <7, resuscitation, prelabor rupture of membranes, and socioeconomic status.

### Perinatal Conditions

Perinatal opioid exposure was associated with an increased risk of perinatal infection (adjusted OR [AOR], 1.62; 95% CI, 1.38-1.90) (Table 2). This finding was consistent when examined by trimester of exposure and in neonates born to women treated with opioids for an OUD. While in the unadjusted model POE was significantly associated with perinatal infection in neonates born to women treated with opioids for pain (OR, 1.51; 95% CI, 1.14-1.99), when adjusted for preterm birth, low birth weight for gestational age, low Apgar score (<7), resuscitation, prelabor rupture of membranes, and socioeconomic status, the association was no longer significant (AOR, 1.10; 95% CI, 0.82-1.46). Common perinatal infections in neonates with POE included sepsis, conjunctivitis, urinary tract infections, and candidiasis.

The AOR for being diagnosed with a perinatal infection was 2.91 times (95% CI, 2.36-3.57) greater in neonates with POE who were also diagnosed with NAS compared with nonexposed neonates. When both POE and NAS were included as separate variables in the model, POE exposure was no longer significantly associated with perinatal infection (OR, 1.12 [95% CI, 0.93-1.35]; AOR, 1.01 [95% CI, 0.84-1.22]), while NAS was associated with a significant increase in perinatal infection (OR, 3.43 [95% CI, 2.93-4.01]; AOR, 2.48 [95% CI, 2.11-2.91]).

Associations between POE and perinatal eczema and dermatitis overall, by trimester, and by indication for dispensing were observed (AOR, 11.91; 95% CI, 9.84-14.41) (Table 2). However, NAS appeared to be a major driver of perinatal eczema and dermatitis in neonates with POE (AOR, 31.11; 95% CI, 24.64-39.28). In models examining both POE and NAS, POE remained significantly associated with perinatal eczema and dermatitis (OR, 3.62 [95% CI, 2.78-4.71]; AOR, 3.89 [95% CI, 3.01-5.02]), as did NAS (OR, 11.24 [95% CI, 8.87-14.25]; AOR, 7.80 [95% CI, 6.18-9.85]).

Perinatal eczema and dermatitis were predominantly diaper dermatitis (2188 neonates [87.7%]) or erythema intertrigo (256 neonates [10.3%]). Per the overall risk of eczema and dermatitis, both POE and NAS were associated with an increased risk of diaper dermatitis (POE: AOR, 3.54 [95% CI, 2.71-4.61]; NAS: AOR, 6.66 [95% CI, 5.24-8.47]) and erythema intertrigo (POE: AOR, 3.15 [95% CI, 1.55-6.40]; NAS: AOR, 4.97 [95% CI, 2.57-9.60]).

### Childhood Conditions Associated With Immune Sensitivity

Prenatal opioid exposure was associated with an increased risk of hospitalization and/or ED presentations for asthma compared with nonexposure (adjusted hazard ratio [AHR], 1.44; 95% CI, 1.16-1.79) (Table 3). This observation was consistent in both the crude and adjusted models overall and by trimester of exposure, NAS diagnosis, and indications for opioid use. Increased risk of hospitalization and/or ED presentation for eczema and dermatitis was observed in children with POE compared with nonexposed children (AHR, 1.35; 95% CI, 1.05-1.74). The increased risk was observed for children exposed to opioids used to treat OUD (AHR, 1.47; 95% CI, 1.08-1.99) but not pain. Prenatal opioid exposure was not associated with an increased risk of hospitalization or ED presentation for allergies and anaphylaxis compared with nonexposure, overall or by subgroups (Table 3).

**Table 3.** Childhood Hospitalization and Emergency Department Presentation for Conditions Associated With Immune Sensitivity or Infection in Children With vs Without POE in Western Australia, 2003-2018

	No. (%)	HR (95% CI)^a^	*P* value	AHR (95% CI)^b^	*P* value
**Asthma**					
No POE	11 110 (2.8)	NA	NA	NA	NA
Any POE	82 (5.0)	1.82 (1.46-2.26)	<.001	1.44 (1.16-1.79)	.001
Trimester 1 POE	60 (5.1)	1.88 (1.46-2.42)	<.001	1.48 (1.15-1.91)	.003
Trimester 2 POE	66 (5.4)	1.99 (1.56-2.54)	<.001	1.56 (1.22-1.98)	<.001
Trimester 3 POE	63 (5.0)	1.85 (1.44-2.37)	<.001	1.45 (1.13-1.86)	.003
POE + NAS	39 (5.9)	2.15 (1.57-2.95)	<.001	1.57 (1.14-2.15)	.005
POE pain	35 (5.2)	1.89 (1.36-2.64)	<.001	1.70 (1.22-2.37)	.002
POE OUD	50 (5.0)	1.82 (1.38-2.40)	<.001	1.33 (1.01-1.76)	.04
**Eczema and dermatitis**					
No POE	9135 (2.3)	NA	NA	NA	NA
Any POE	60 (3.6)	1.60 (1.24-2.06)	<.001	1.35 (1.05-1.74)	.02
Trimester 1 POE	38 (3.2)	1.43 (1.04-1.97)	.03	1.21 (0.88-1.66)	.25
Trimester 2 POE	42 (3.4)	1.52 (1.12-2.06)	.007	1.28 (0.94-1.73)	.12
Trimester 3 POE	45 (3.6)	1.59 (1.19-2.13)	.002	1.34 (1.00-1.80)	.05
POE + NAS	24 (3.6)	1.59 (1.07-2.37)	.02	1.27 (0.85-1.90)	.24
POE pain	18 (2.7)	1.16 (0.73-1.85)	.52	1.08 (0.68-1.71)	.75
POE OUD	42 (4.2)	1.85 (1.37-2.51)	<.001	1.47 (1.08-1.99)	.01
**Allergy and anaphylaxis**					
No POE	19 196 (4.8)	NA	NA	NA	NA
Any POE	75 (4.5)	1.02 (0.82-1.28)	.84	1.08 (0.86-1.36)	.49
Trimester 1 POE	45 (3.8)	1.06 (0.79-1.43)	.68	1.13 (0.84-1.52)	.40
Trimester 2 POE	48 (3.9)	1.10 (0.83-1.46)	.53	1.17 (0.88-1.55)	.29
Trimester 3 POE	51 (4.0)	0.99 (0.75-1.30)	.92	1.05 (0.79-1.38)	.75
POE + NAS	20 (3.0)	0.80 (0.52-1.24)	.32	0.86 (0.55-1.33)	.50
POE pain	40 (5.9)	1.05 (0.77-1.44)	.74	1.10 (0.81-1.50)	.54
POE OUD	35 (3.5)	0.99 (0.71-1.38)	.96	1.06 (0.76-1.49)	.72
**Infection**					
No POE	226 049 (56.5)	NA	NA	NA	NA
Any POE	1044 (63.0)	1.28 (1.21-1.36)	<.001	1.18 (1.11-1.26)	<.001
Trimester 1 POE	700 (59.7)	1.17 (1.09-1.26)	<.001	1.08 (1.00-1.16)	.04
Trimester 2 POE	745 (61.1)	1.21 (1.13-1.30)	<.001	1.11 (1.04-1.20)	.003
Trimester 3 POE	783 (61.9)	1.25 (1.17-1.34)	<.001	1.16 (1.08-1.24)	<.001
POE + NAS	424 (63.7)	1.32 (1.20-1.45)	<.001	1.19 (1.08-1.31)	<.001
POE pain	473 (69.7)	1.50 (1.37-1.64)	<.001	1.44 (1.32-1.58)	<.001
POE OUD	591 (58.7)	1.15 (1.06-1.25)	<.001	1.04 (0.96-1.12)	.38

Abbreviations: AHR, adjusted hazard ratio; HR, hazard ratio; NA, not applicable; NAS, neonatal abstinence syndrome; OUD, opioid use disorder; POE, prenatal opioid exposure.

^a^ Compared with no exposure.

^b^ Adjusted for maternal age, sex, cesarean delivery, smoking status during pregnancy, and preterm birth.

### Childhood Infections

Prenatal opioid exposure was associated with an increased risk of infection overall and by trimester of exposure, although in general, the increased risk was modest (8%-18%) (Table 3). Prenatal opioid exposure as a result of opioids used to treat pain was associated with a 45% increase in risk of infection (AHR, 1.44; 95% CI, 1.32-1.58), while POE associated with opioids used to treat OUD was only associated with a 4% increased risk of infection (AHR, 1.04; 95% CI, 0.96-1.12).

The association between POE and infection was not consistent for all infection types (Table 4). In the crude analysis, POE was associated with an increased risk of enteric infections, neurologic and eye infections, upper respiratory tract infections, lower respiratory tract infections, digestive tract infections, skin and soft tissue infections, and other infections (eg, scabies, nonspecific lymphadenitis, and viral infection). However, following adjustment for maternal age, child sex, cesarean delivery, smoking status during pregnancy, and preterm birth, this association was limited to enteric infections, lower respiratory tract infections, and other infections.

**Table 4.** Infection Types in Children With vs Without Prenatal Opioid Exposure

Infection type	No. (%)		HR (95% CI)	*P* value	AHR (95% CI)^a^	*P* value
	No exposure	Any exposure				
Enteric	63 016 (15.8)	319 (19.3)	1.25 (1.12-1.39)	<.001	1.20 (1.08-1.34)	.001
Bloodstream	2208 (0.6)	13 (0.8)	1.43 (0.83-2.46)	.20	1.06 (0.61-1.83)	.85
Neurologic and eye	10 165 (2.5)	62 (3.7)	1.49 (1.16-1.91)	.002	1.24 (0.96-1.59)	.09
Upper respiratory tract	122 163 (30.6)	546 (33.0)	1.12 (1.03-1.22)	.009	1.06 (0.98-1.16)	.16
Lower respiratory tract	51 793 (13.0)	316 (19.1)	1.53 (1.37-1.71)	<.001	1.18 (1.05-1.31)	.004
Digestive tract	12 131 (3.0)	67 (4.1)	1.35 (1.06-1.71)	.02	1.22 (0.96-1.56)	.10
Genitourinary	17 324 (4.3)	75 (4.5)	1.05 (0.84-1.32)	.68	1.05 (0.83-1.32)	.68
Skin and soft tissue	18 904 (4.7)	118 (7.1)	1.53 (1.28-1.83)	<.001	1.12 (0.93-1.34)	.24
Other^b^	130 288 (32.6)	668 (40.3)	1.33 (1.23-1.43)	<.001	1.23 (1.14-1.33)	<.001

Abbreviations: AHR, adjusted hazard ratio; HR, hazard ratio.

^a^ Adjusted for maternal age, child sex, cesarean delivery, smoking status during pregnancy, and preterm birth.

^b^ Includes scabies, nonspecific lymphadenitis, and viral infection.

Further examination of the association between indication for opioid use and specific types of infections revealed no increased risk of any type-specific infection associated with POE from opioids for the treatment of OUD. However, POE secondary to opioids prescribed to treat pain was associated with an increased risk of enteric infections, neurological and eye infections, upper respiratory tract infections, lower respiratory tract infections, and other infections (Table 5).

**Table 5.** Infection Types in Children With and Without Prenatal Opioid Exposure Stratified by Opioids Used to Treat Pain and OUD

Infection type	Opioids for pain			Opioids for OUD		
	No. (%)	AHR (95% CI)^a^	*P* value	No. (%)	AHR (95% CI)^a^	*P* value
Enteric	155 (22.8)	1.51 (1.29-1.77)	<.001	169 (16.8)	1.00 (0.86-1.17)	.97
Bloodstream	6 (0.9)	1.29 (0.58-2.87)	.54	7 (0.7)	0.88 (0.42-1.86)	.75
Neurologic and eye	31 (5.6)	1.70 (1.20-2.42)	.003	33 (3.3)	1.00 (0.71-1.41)	.99
Upper respiratory tract	264 (38.9)	1.34 (1.19-1.51)	<.001	295 (30.3)	0.90 (0.80-1.01)	.08
Lower respiratory tract	137 (20.2)	1.40 (1.18-1.66)	<.001	185 (18.4)	1.04 (0.90-1.21)	.56
Digestive tract	26 (3.8)	1.25 (0.85-1.83)	.27	42 (4.2)	1.20 (0.89-1.63)	.23
Genitourinary	34 (5.0)	1.18 (0.84-1.66)	.33	42 (4.2)	0.95 (0.70-1.29)	.74
Skin and soft tissue	42 (6.2)	1.22 (0.90-1.66)	.19	77 (7.7)	1.05 (0.84-1.31)	.69
Other^b^	307 (45.2)	1.48 (1.32-1.65)	<.001	374 (37.1)	1.09 (0.98-1.21)	.10

Abbreviations: AHR, adjusted hazard ratio; OUD, opioid use disorder.

^a^ Adjusted for maternal age, sex, cesarean delivery, smoking status during pregnancy, and preterm birth.

^b^ Includes scabies, nonspecific lymphadenitis, and viral infection.

### Autoimmune Conditions

Prenatal opioid exposure was not associated with an increased risk of autoimmune conditions, although autoimmune conditions were overall uncommon in our patient population. In the POE group, fewer than 5 children (<0.3%) were hospitalized or presented to the ED before the age of 5 years with an autoimmune disease compared with 1335 (0.3%) in the nonexposed group.

## Discussion

In this cohort study, POE was associated with an increase in the incidence of hospitalizations and ED presentations for immune-related conditions in Western Australia. However, there was a degree of variability among conditions. Additionally, the presence of NAS and the type of opioids involved may have contributed to the association between POE and specific immune-related conditions. The results have important implications, as prenatal exposure to prescription opioids is estimated to occur in up to 14.1% of pregnancies,^26,27^ while NAS has been found in up to 0.8% of neonates.^18,28,29,30^

Prenatal opioid exposure was associated with an increased risk of infection and eczema and dermatitis during the perinatal period. For both conditions, NAS was observed to play an important role in the increased risk. Prior research has suggested that at birth, neonates with POE may have substantially reduced neutrophils, decreased levels of inflammatory cytokines in the serum, and reduced IL-2 production during in vitro CD4^+^ T-cell proliferation compared with control neonates.^12^ It has been suggested that this immune profile could promote susceptibility to infection, which is consistent with the observation in our study. However, in the study of inflammatory markers, the investigators did not differentiate between neonates with and without NAS, although in a separate cohort, they found that NAS was also associated with a deficiency in neutrophils.

The increased risk of eczema and dermatitis in the perinatal period in neonates with POE was consistent with research by Arter et al.^31^ They found an increased risk of diaper dermatitis in neonates with POE compared with nonexposed neonates. Additionally, they observed rates of diaper dermatitis in infants with POE and NAS that were approximately twice that of infants with only POE. With the implementation of simple skin care regimens within the hospital, the incidence of diaper dermatitis can be substantially reduced.^32^ Eczema and dermatitis rarely result in hospitalization; thus, the increased risk could also be the result of the increased length of time neonates with POE typically spend in the hospital following birth, making it easier for the condition to be observed in the hospital.

Beyond the perinatal period, POE was associated with a modest 35% increase in the odds of being diagnosed with eczema and dermatitis (compared with a >11-fold increase in the perinatal period). Interestingly, the increases did not appear to be associated with NAS, as it was in the perinatal period. The risk of eczema and dermatitis was significantly higher in children with POE associated with opioids used for treatment of OUD (methadone or buprenorphine) compared with nonexposed children. However, the same was not found for children with POE associated with opioids used to treat pain (predominantly oxycodone, codeine, and morphine). The opioidergic system has been found to play a role in pruritus and potentially eczema. Bigliardi-Qi et al^33^ found that mu-opioid receptors were downregulated in skin biopsy samples of patients with eczema. Tominaga et al^34^ were unable to replicate such results but instead found downregulation of the kappa-opioid receptors in the epidermis. The potential role of kappa-opioid receptors in eczema may explain the difference in the association for opioids used to treat pain and OUD. Unlike many other opioids, buprenorphine is a κ-opioid antagonist. Potentially, prenatal exposure to buprenorphine may influence the sensitivity or distribution of κ-opioid receptors in the epidermis, increasing an individual’s susceptibility to eczema. Alternatively, the difference in association may reflect a difference in the pregnant women within each of the study groups in terms of other risk factors.

Prenatal opioid exposure was associated with an increased risk of hospitalization and ED presentation for asthma compared with nonexposure. This association was not observed to be modified by the trimester of exposure, NAS diagnosis, or the type of opioids involved. Shaheen et al^35^ also observed an association between POE and an increased odds of childhood asthma and wheezing (AOR, 1.39; 95% CI, 1.30-1.49). However, they suggested that it was not a true association but, rather, a result of confounding by an unmeasured factor, such as chronic pain or anxiety. Despite the association between POE and asthma (a common allergic reaction in children), POE was not associated with an increased risk of hospitalization and ED presentation for allergies and anaphylaxis.^36^ The identification of allergic and anaphylaxis events via hospital and ED records may only capture the most severe cases, with mild and moderate cases of allergies largely missed. Thus, while we found no association at the severe end of the spectrum of such conditions, further research is warranted on the association with less severe allergies.

A modest (18%) increase in infection was observed in neonates with POE compared with unexposed neonates, but this was associated with exposure to opioids used to treat pain (significant 45% increase) rather than opioids used to treat OUD (nonsignificant 4% increase). This finding was consistent upon examination of type-specific infections, with exposure to opioids used to treat pain associated with a significant increase in the risk of enteric infections, neurologic or eye infections, upper respiratory infections, lower respiratory infections, and other infections compared with no exposure. In comparison, POE associated with OUD medications was not associated with an increased risk of any type-specific infection.

The presence of an association between opioids used to treat pain and infection, but not opioids used to treat OUD, appears paradoxical, especially given the risk of maternal transmission of infections, such as hepatitis, that are common in women with an OUD.^37^ This finding highlights the need to examine opioid safety in pregnancy by individual opioid types. The characteristics and behaviors of women being treated with opioids for pain differ from those being treated for OUD.^38^ Additionally, the drugs used for each condition differ in terms of their pharmacokinetics and pharmacodynamics, as do the dosages used, which can contribute to differences in outcomes. It is not known how the differential pharmacology is associated with fetal immune system development or if there is a difference in inflammatory response.

There was no evidence to suggest an increased risk of autoimmune conditions in children with POE. However, these conditions are rare and are commonly diagnosed later in childhood. Thus, further investigation of the association of POE and autoimmune conditions would require a larger sample size and longer follow-up.

### Limitations

The naturalistic study design is subject to a number of limitations. Importantly, the study only examined exposure to Schedule 8 opioids and, thus, did not capture exposure to illicit opioids or lower schedule opioids. Similarly, we were only able to identify the diagnosis of conditions in which the child was admitted to the hospital or presented to the ED. As such, conditions diagnosed in the community would have been missed. Thus, this study may reflect more severe cases or cases that have not been suitably treated in the community (eg, treated by general practitioners and pediatricians).

Examination of trimester exposure did not produce strong indications of a specific period of exposure being associated with the outcomes of interest. However, as we defined exposure as the dispensing of 2 or more prescriptions for opioids, exposure typically occurred over multiple trimesters, making it difficult to elicit whether specific periods of immune development may be more sensitive to opioid exposure.

## Conclusions

In this cohort study, the findings suggest that POE may modify the developing immune system, changing how the immune system responds to subsequent conditions. However, we observed a substantial difference between conditions and opioids used for specific indications, including the treatment of pain and OUD. The presence of NAS may play an important role in the risk of perinatal conditions, including infection and eczema and dermatitis. However, following the perinatal period, NAS may not be a primary driver of immune-related conditions.

## Notes

Supplement.
Data Sharing Statement
