# Supplementary material for: Prenatal Opioid Exposure and Immune-Related Conditions in Children
Source: JAMA Netw Open. 2024 Jan 17;7(1):e2351933. doi: 10.1001/jamanetworkopen.2023.51933 (PMC10794935; doi:10.1001/jamanetworkopen.2023.51933)
Supplement: Supplement. — Data Sharing Statement [file jamanetwopen-e2351933-s001.pdf]

Data Sharing Statement

Kelty. Prenatal Opioid Exposure and Immune-Related Conditions in Children. *JAMA Netw Open*. Published January 17, 2024. doi:10.1001/jamanetworkopen.2023.51933

Data
Data available: No

Additional Information
Explanation for why data not available: It was a condition of our access to the data that it would not be shared.
